# Rapid Chondrolysis After Lateral Meniscal Repair and Anterior Cruciate Ligament Reconstruction Combined With Segond Fracture Fragment Fixation: A Case Report

**DOI:** 10.7759/cureus.76872

**Published:** 2025-01-03

**Authors:** Rishav Pokharel, Takehiko Matsushita, Kyohei Nishida, Yuta Nakanishi, Ryosuke Kuroda

**Affiliations:** 1 Department of Orthopedic Surgery, Kobe University Hospital, Kobe, JPN; 2 Department of Orthopedic Surgery, Kobe University Graduate School of Medicine, Kobe, JPN

**Keywords:** meniscal repair, meniscus extrusion, radial tear, rapid chondrolysis, segond fracture

## Abstract

We present a case of rapid chondrolysis after the repair of a lateral meniscus (LM) radial tear. An 18-year-old male competitive skier sustained an anterior cruciate ligament (ACL) injury associated with a Segond fracture and LM radial tear in the posterior segment. The patient underwent a two-stage surgery, LM repair and internal fixation of the fracture fragment, followed by ACL reconstruction. Although the postoperative course was uneventful, the patient experienced persistent joint effusion after starting ski practice six months postoperatively. An arthroscopic examination was performed one year after ACL reconstruction, and severe cartilage damage was observed in the lateral femoral condyle and tibial plateau, whereas complete healing of the repaired LM was observed. Despite complete arthroscopic healing of the meniscus, meniscal function may not be fully restored by isolated meniscal repair of radial tears associated with ACL injury. The development of new surgical methods may be necessary to improve functional restoration.

## Introduction

Meniscal injuries frequently coincide with sports activities, either in conjunction with ligament injuries or in isolation [1,2]. The menisci play a crucial role in distributing load, absorbing shock, and stabilizing the knee joint. When injured, the biomechanics of the knee are altered, leading to increased stress on the articular cartilage and subsequent degeneration [3,4]. Therefore, preserving meniscal integrity is important for preventing cartilage damage and slowing the progression of osteoarthritis.

Meniscal tears are classified as longitudinal, horizontal, radial, or complex, with radial tears extending perpendicularly from the inner avascular zone of the meniscus toward the outer peripheral zone. Radial tears disrupt the circumferential fibers of the meniscus, which are responsible for providing structural integrity and stability to the meniscus, leading to loss of hoop function. Historically, meniscectomy has been performed to treat meniscal radial tears, considering the low healing potential of the inner avascular portion of the meniscus. Previous studies have documented the phenomenon of rapid chondrolysis following lateral meniscectomy, which is characterized by the accelerated degeneration of the articular cartilage that occurs shortly after the surgical removal of the meniscus [5-7]. Thus, meniscal repair is increasingly being recognized as the preferred treatment for radial meniscal tears to preserve the meniscus. Although good clinical outcomes have been reported after meniscal repair for radial tears [8], several studies have reported unfavorable outcomes and frequent incomplete healing of the repaired meniscus [9,10], suggesting that the function of the repaired meniscus is not fully restored.

Here, we report a case of rapid chondrolysis after the repair of a lateral meniscus (LM) radial tear associated with an anterior cruciate ligament (ACL) injury and a Segond fracture despite achieving complete arthroscopic healing.

## Case presentation

**Figure 1:**
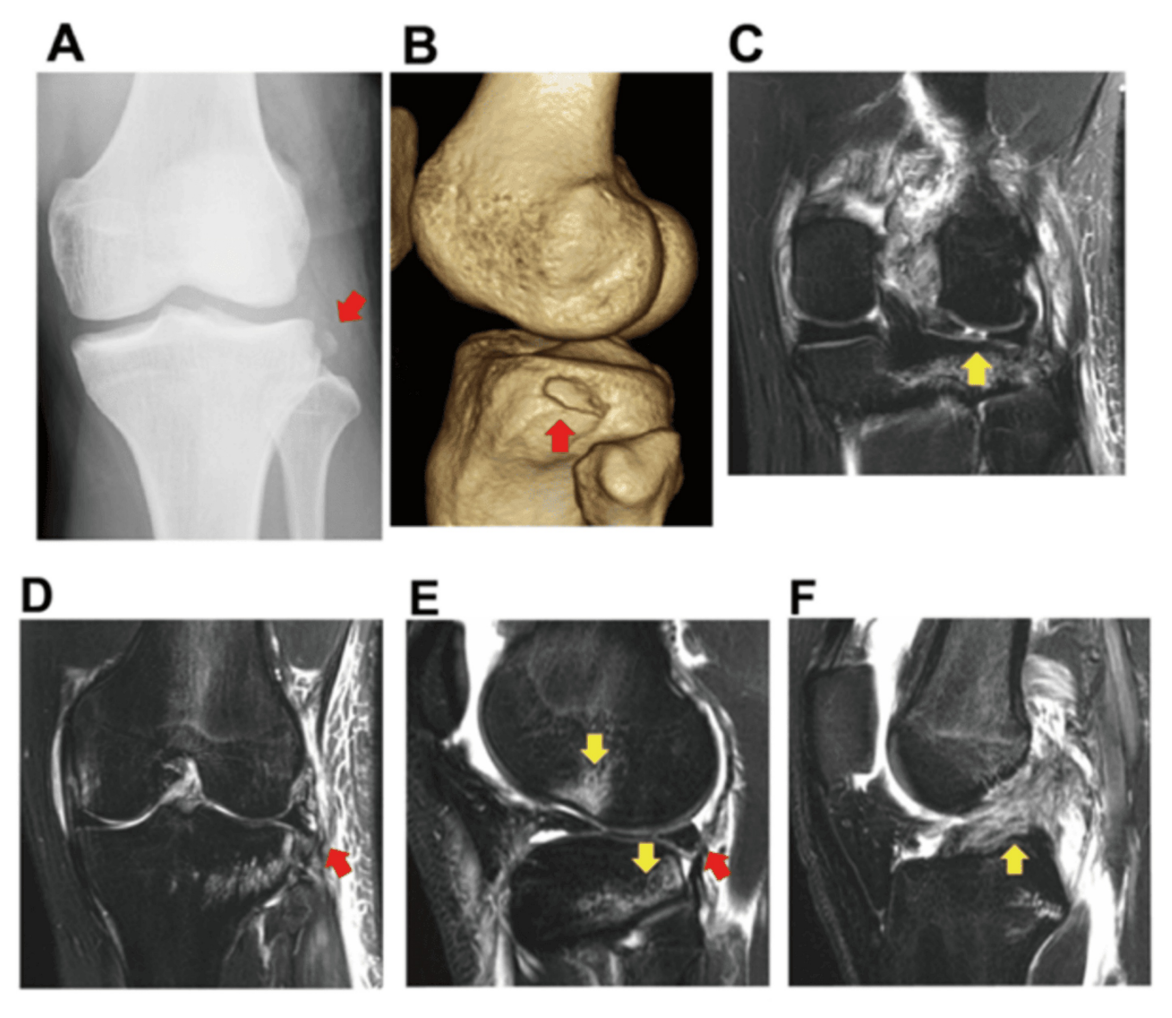
Preoperative images. (A) Anteroposterior radiograph. The red arrow indicates the Segond fracture fragment. (B) The three-dimensional CT image showing the Segond fracture fragment (red arrow). (C) The coronal view MR image showing the lateral meniscal tear in the posterior segment (yellow arrow). (D) The Segond fracture site associated with lateral extrusion of the lateral meniscus and a detachment of the joint capsule (red arrow). (E) The sagittal view MR image showing the posterior extrusion of the lateral meniscus (red arrow) and a significant bone bruise-like high-signal area on both tibial and femoral sides (yellow arrows). (F) The sagittal view MR image showing the ACL injury at the femoral attachment site (yellow arrow). CT: computed tomography; MR: magnetic resonance; ACL: anterior cruciate ligament

An 18-year-old competitive skier presented to an emergency room with an acute knee injury, showing severe left knee pain and a restricted range of motion (ROM) following a competitive game. On initial examination of the left knee, ROM was limited to 5-40 degrees, and the Lachman test result was positive, while the pivot-shift test could not be performed due to pain. An anteroposterior radiograph revealed a Segond fracture (Figure 1A). The computed tomography (CT) scan of the knee showed a 7.7×18.4 mm size slightly displaced avulsion fracture fragment in the proximal portion of the lateral tibial condyle (Figure 1B). Magnetic resonance imaging (MRI) showed a radial tear in the posterior segment of the LM (Figure 1C) associated with a 4.7 mm lateral extrusion of the meniscus and a detachment of the joint capsule and the avulsed fragment (Figure 1D). The sagittal view MRI also showed a 6.6 mm posterior extrusion of the LM and a significant bone bruise-like high-signal area (Figure 1E). Furthermore, an ACL injury was noted on the femoral attachment side (Figure 1F).

Considering the size of the avulsed fragment and the condition of the LM, a two-stage surgery was planned, with the first surgery involving an arthroscopic examination performed seven days after the injury.

**Figure 2:**
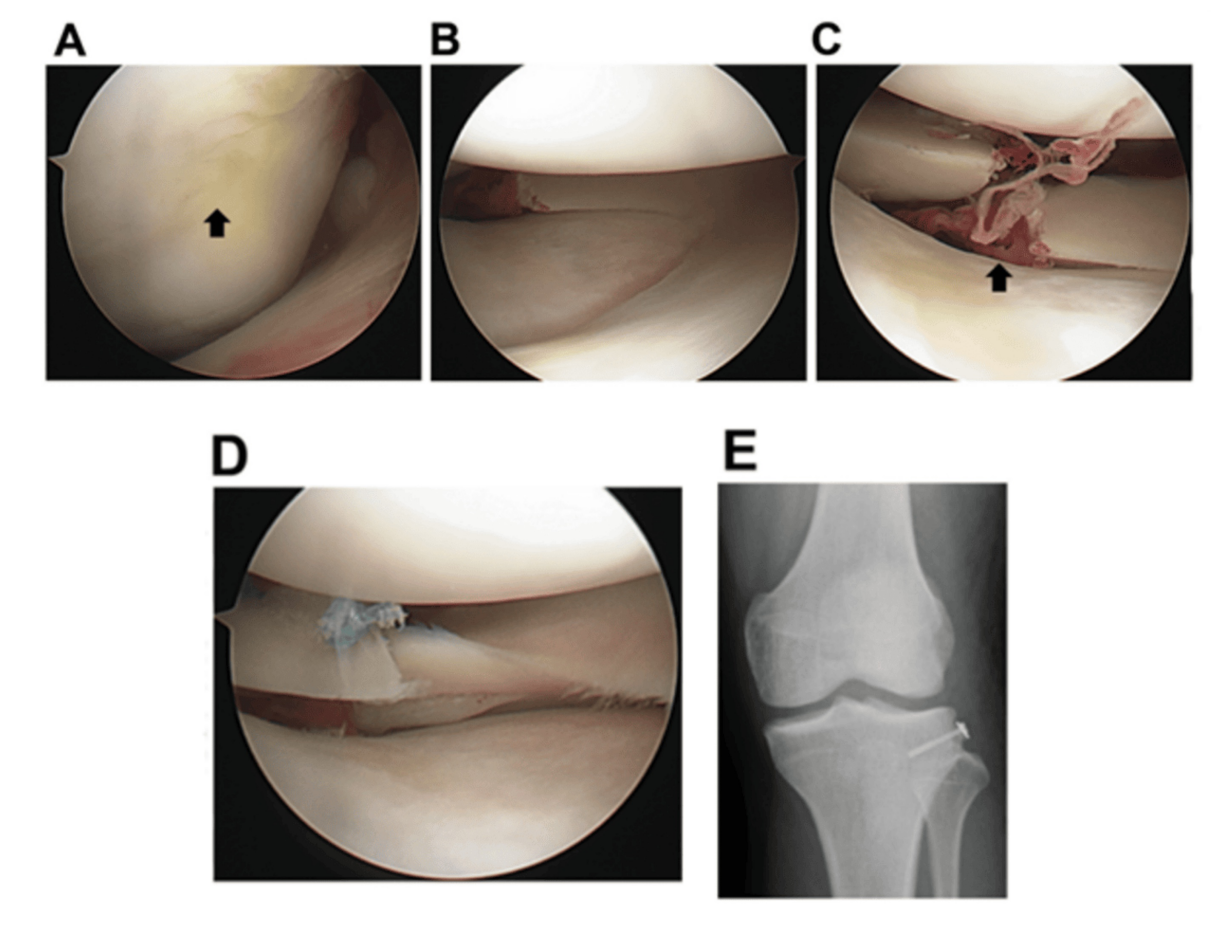
Arthroscopic view images at the time of lateral meniscal repair. (A) ICRS grade I cartilage injury in the weight-bearing area of the femoral side at full extension (black arrow). (B) An arthroscopic view showing no obvious cartilage damage in the tibial side. (C) A complete radial tear of the posterior segment (black arrow). (D) After side-to-side repair of the tear. (E) Anteroposterior view radiograph after the osteosynthesis. ICRS: International Cartilage Repair Society

Arthroscopic examination confirmed the complete rupture of the ACL. A 10×10 mm International Cartilage Repair Society (ICRS) grade I cartilage injury was observed in the weight-bearing area of the femoral side at full extension (Figure 2A), whereas no obvious cartilage damage was found in the tibia (Figure 2B). Additionally, a complete radial tear was observed in the posterior segment of the LM (Figure 2C).

For the lateral meniscal radial tear, side-to-side repair was performed using two 2-0 Fiberwire sutures (Arthrex Inc., Naples, Florida, United States) with an all-inside technique (Figure 2D). Open reduction and internal fixation was performed for the Segond fracture fragments with the fragment being fixed using a 3 mm half-thread cannulated lag screw (Figure 2E). ROM exercise and partial weight-bearing were initiated seven days and 14 days after surgery, respectively. ROM of the operated knee was recovered to 0 degrees in extension and 130 degrees in flexion six weeks after surgery.

**Figure 3:**
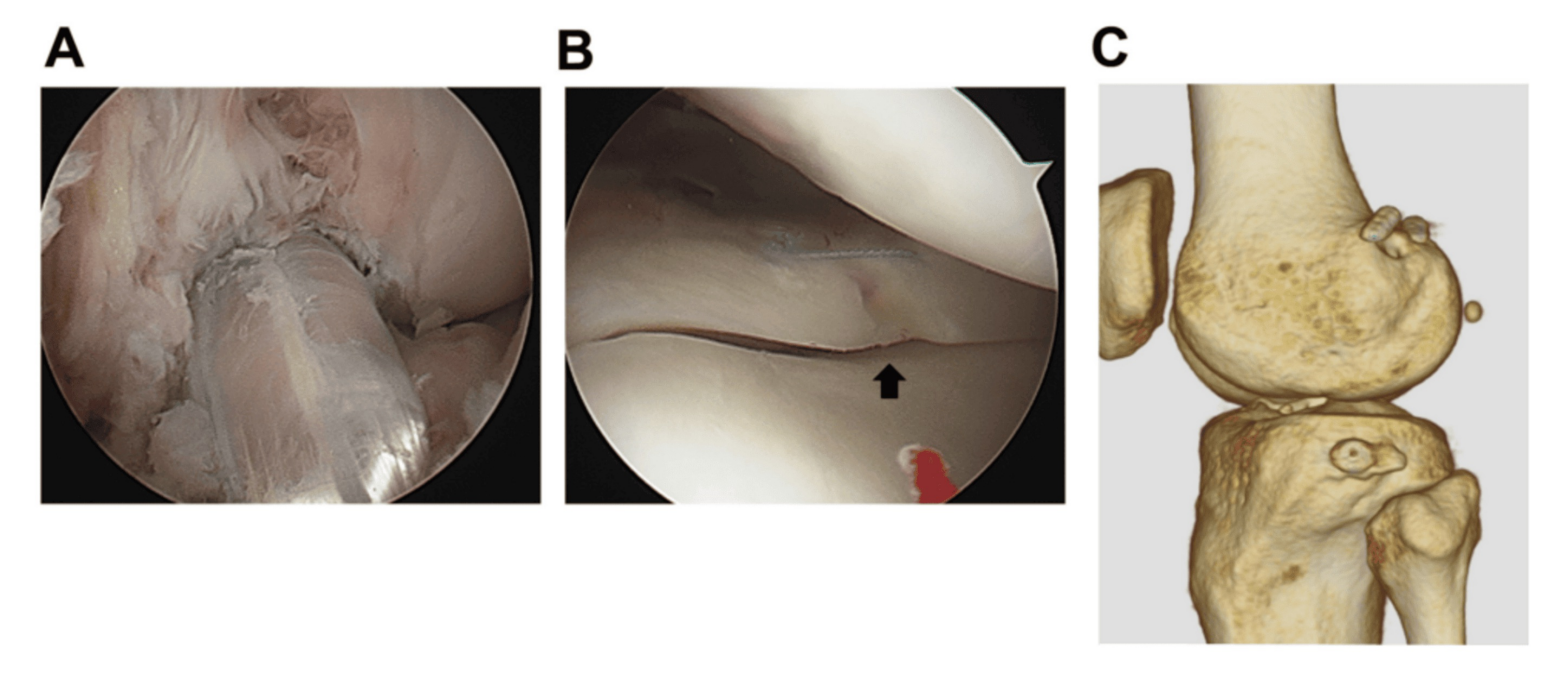
Arthroscopic view images at the time of ACL reconstruction and the postoperative CT image. (A) After the double-bundle ACL reconstruction with a hamstring tendon (anteromedial portal view). (B) The repaired site of the lateral meniscus (black arrow). (C) The postoperative three-dimensional CT image. ACL: anterior cruciate ligament; CT: computed tomography

The second surgery was performed six weeks after the first surgery, during which double-bundle ACL reconstruction was performed using a hamstring autograft (Figure 3A). Partial healing of the LM was observed at the time of ACL reconstruction (Figure 3B), and the fixed fragment was stable at the reduced site (Figure 3C).

**Figure 4:**
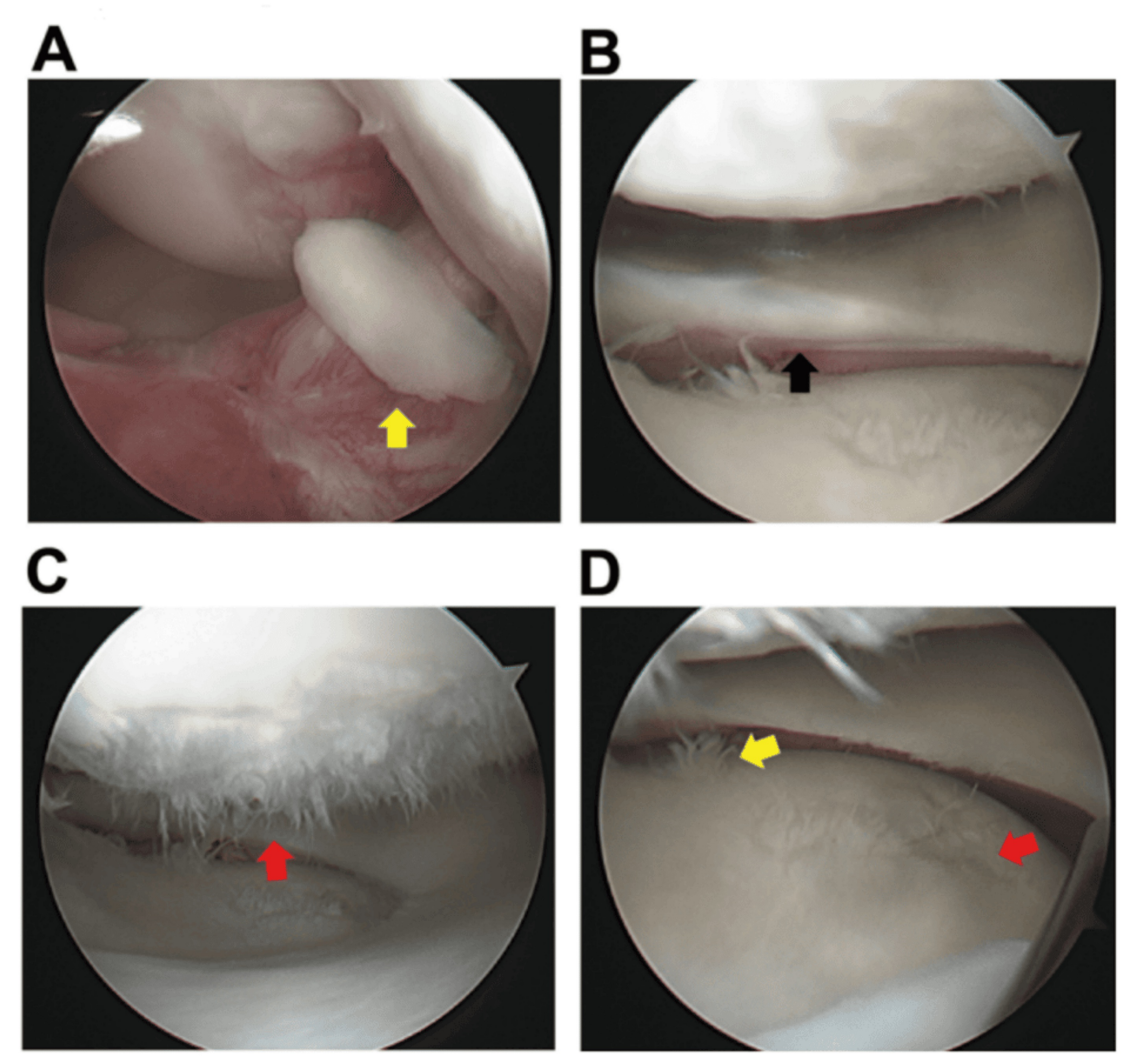
Arthroscopic view images at the time of debridement. (A) A free cartilage fragment in front of the reconstructed ACL (yellow arrow). (B) Arthroscopically completely healed site with a scar tissue-like condition (black arrow). (C) The severely damaged cartilage of the femoral condyle (red arrow). (D) The extruded lateral meniscus. Cartilage damage was observed in the middle lesion of the tibial plateau uncovered by the extruded meniscus (red arrow), while the cartilage damage was also observed in the posterior side (yellow arrow). ACL: anterior cruciate ligament

ROM exercise and partial weight-bearing were initiated two days after the second surgery, full weight-bearing was permitted at postoperatively two weeks, and jogging was initiated at postoperatively four months. The postoperative course was uneventful, and the patient started alpine ski practice six months after ACL reconstruction. However, the patient repeatedly experienced swelling and pain approximately three months after initiating ski practice. Joint aspiration was performed two times, and serous yellowish synovial fluid without any sign of infection was obtained. Regarding knee stability, the Lachman and pivot-shift tests were negative, and the side-to-side difference in anteroposterior tibial translation on the KT-2000 was 1 mm. The patient's condition was regularly monitored using an activity control. However, the effusion and pain persisted, and a third operation was performed one year after the second operation. A post screw was removed at the time of the arthroscopic examination. Arthroscopically, the reconstructed ACL was well covered by synovial tissue, and good tension of the graft was confirmed by probing. The repaired LM healed completely with a scar tissue-like condition (Figure 4A). However, numerous cartilage fragments and severely damaged cartilage injuries were observed in the femoral condyle and tibial plateau (Figure 4B-4D). The cartilage injury states were classified as ICRS grades II and III for the femoral condyle and tibial plateau, respectively, while there was no cartilage damage in other areas. Meniscal extrusion was observed in the middle segment (Figure 4D), and the detached cartilage fragments were subsequently debrided.

**Figure 5:**
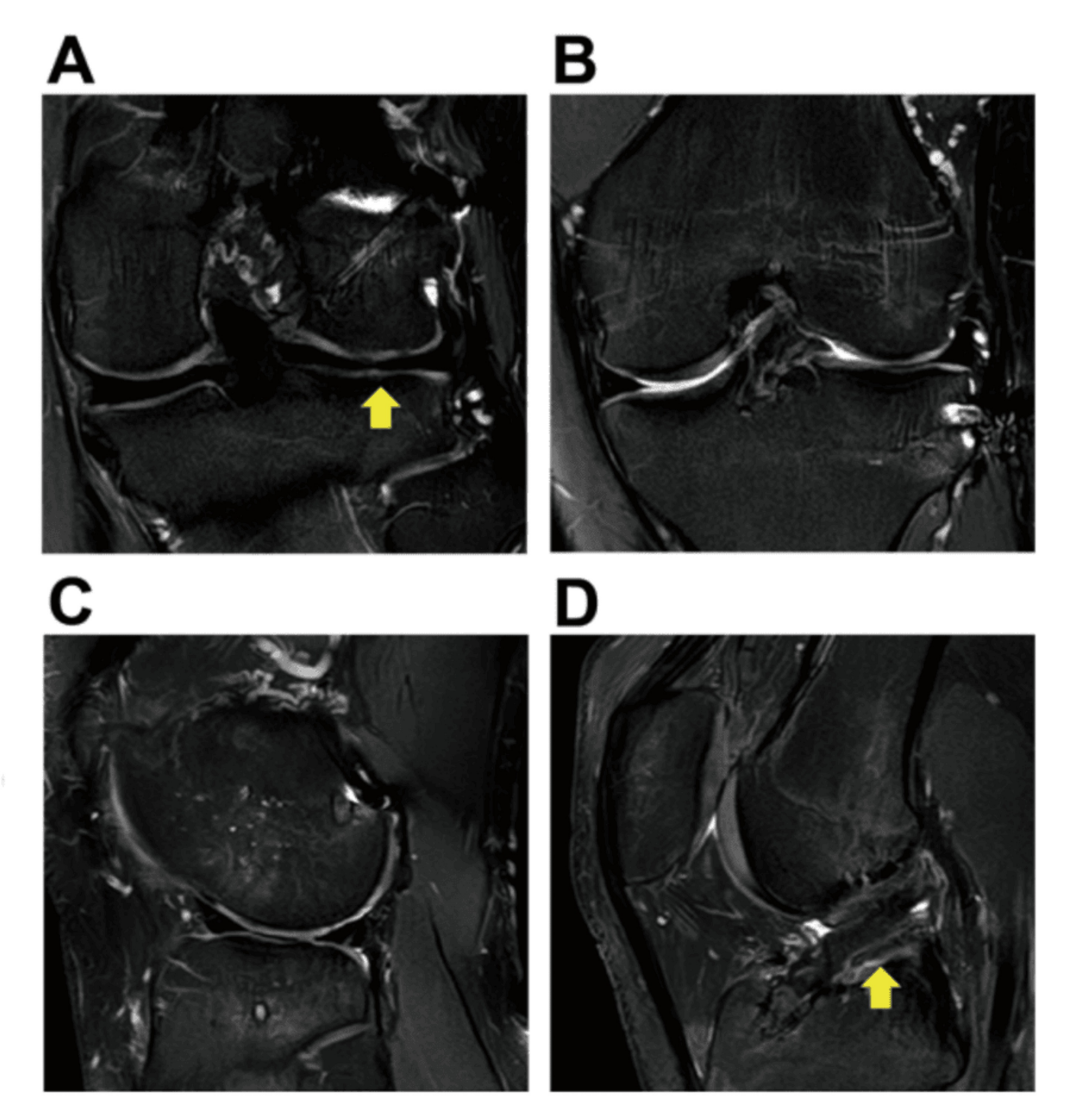
MR images two years after ACL reconstruction. (A) A coronal view slice showing the well-repaired posterior segment of the lateral meniscus (yellow arrow). (B) A coronal view slice showing the slightly extruded middle segment of the lateral meniscus adjacent to the fixed fracture site. (C) A sagittal view slice showing no obvious posterior extrusion of the lateral meniscus. (D) A sagittal view slice showing the reconstructed ACL (yellow arrow). MR: magnetic resonance; ACL: anterior cruciate ligament

At the two-year follow-up, the posterior segment of LM was healed without a gap, while a 2.2 mm lateral extrusion of the middle segment of LM was observed on the coronal view MRI (Figure 5A, 5B). Posterior extrusion of LM was minimal with 0.7 mm on the sagittal view (Figure 5C), and the reconstructed ACL was detected without an obvious abnormal signal (Figure 5D). After the third surgery, the patient did not return to the original competitive level, although the symptoms improved.

## Discussion

We present a case of rapid chondrolysis after lateral meniscal repair and ACL reconstruction combined with Segond fracture fragment fixation.

Radial tears in the meniscus compromise essential functions, including load distribution, shock absorption, and joint stability. Therefore, early detection and appropriate management to preserve the meniscus are crucial for preserving knee joint function [11]. Krych et al. investigated the meniscal tear patterns in a total of 600 patients with an acute ACL injury and highlighted the incidence of posterior horn lateral meniscal oblique radial tears (LMORT) associated with ACL injuries [12]. For the treatment of LMORT, Zhuo et al. examined the outcomes after arthroscopic side-to-side repair for complete radial root tears of the posterior LM and reported that complete healing was observed in 86.4% of the patients at the time of second-look arthroscopy [13]. Similarly, Jeon et al. investigated the incidence of LMORT and healing status after LMORT repair and reported that complete healing of LMORT was achieved in 80.3% of the patients who underwent second-look arthroscopy [14]. The results of these reports are in line with those of the present case and suggest that the posterior LM has high healing potential. However, in the present case, severe cartilage damage was observed despite complete healing. This observation also suggested that the biomechanical function of the repair was not completely restored. Tsujii et al. compared the healing status of the repaired meniscus and the chondral status at the time of second-look arthroscopy in patients who underwent repair of radial/flap tears of the posterior LM combined with ACL reconstruction and those who underwent isolated ACL reconstruction. They found that cartilage damage in the lateral tibial plateau significantly worsened at a mean of 3.4 years postoperatively in patients with posterior LM radial tears compared with those without meniscal tears [9]. Therefore, these observations, together with findings from our case, suggest that the biomechanical function of the meniscus may not be restored fully even if arthroscopically "good healing" is obtained after the repair of the posterior LM radial tears and caution is necessary when treating LM radial tears.

Regarding the mechanism of cartilage damage in the present case, knee instability, cartilage injury in the femoral condyle, and the patient's specific activities, such as being a competitive skier, could all contribute to the cause of severe cartilage damage. However, sufficient knee stability was confirmed by a negative pivot-shift, and no obvious residual instability was detected under anesthesia at the time of arthroscopic debridement. Additionally, newly developed cartilage damage was observed at the distal posterior (weight-bearing area in knee flexion) and was different from the anterior location of the cartilage injury associated with ACL injury, suggesting that different injury mechanisms were involved. Furthermore, complete healing was observed during the arthroscopic examination one year after ACL reconstruction, suggesting that incomplete healing of the meniscus was not responsible for the cartilage damage. Meanwhile, lateral meniscal extrusion was observed at the time of arthroscopic debridement, and the location of the cartilage damage in the tibia was adjacent to the meniscal extrusion. Therefore, early return to high-demand sports activity with the condition of residual lateral meniscal extrusion may have caused the rapid chondrolysis in the present case. Tsujii et al. investigated meniscal extrusion after LM repair combined with ACL in patients with radial and longitudinal LM tears and intact LM, observing posterior extrusion in the radial and longitudinal tear groups compared to the intact meniscus group [15]. Therefore, this study suggests that posterior extrusion is one of the causes of cartilage damage. Although sagittal extrusion was not evident after surgery in our case, these reports indicate that meniscal extrusion is a key pathological condition after radial tear repair. The difference in the direction of meniscal extrusion may be related to meniscocapsular injury associated with Segond fracture, as lateral meniscal extrusion was observed near the Segond fracture site. Although meniscal extrusion can be caused by the elongation of the meniscus or Segond fracture associated with damaged capsular structures including the meniscotibial ligament or the high-demanding alpine ski, it appears to be, at least in part, involved in rapid chondrolysis.

Regarding the surgical method for meniscal radial tears, the optimal surgical method for LM radial tears remains unknown at the moment, and particularly radial tears in the posterior segment remain challenging. Once the circumferential fibers are disrupted by radial tears, it is difficult to fully restore the original biomechanical function using conventional suture techniques which are unlikely to successfully prevent the meniscus elongation and restore the fiber orientation. Additional biological augmentations, such as plate-rich plasma and mesenchymal cells, or biomechanical augmentations, such as centralization, capsulodesis, and meniscal circumferential fiber augmentation [16], may be considered to improve the functional restoration of the meniscus, while further studies on the mechanisms underlying chondrolysis are required.

## Conclusions

Rapid chondrolysis was observed after meniscal repair for the radial meniscal tear and ACL reconstruction combined with Segond fracture fragment fixation, despite complete arthroscopic healing of the meniscus. The findings suggest that the conventional suture method for radial meniscal tears may not fully restore the biomechanical function of the meniscus. Developing new surgical methods and exploring adjunctive therapies are necessary to improve the functional restoration of the meniscus after LM radial tears.
